# Differential impact of host-related factors on survival outcomes between IO+IO and IO+TKI in metastatic renal cell carcinoma: a multicenter retrospective study

**DOI:** 10.1007/s10147-026-03075-2

**Published:** 2026-06-11

**Authors:** Koichi Sugimoto, Shingo Toyoda, Lan Inoki, Keiichiro Mori, Takafumi Yanagisawa, Keita Tamura, Takuhisa Nukaya, Ryoichi Maenosono, Hirofumi Morinaka, Shingo Nishimura, Kensuke Bekku, Kazumasa Komura, Kiyoshi Takahara, Teruo Inamoto, Haruhito Azuma, Kazutoshi Fujita

**Affiliations:** 1 Department of Urology, Kindai University Faculty of Medicine, 3-1-14 Mihara-dai, Minami-ku, Sakai City, 589-8511 Osaka Japan; 2 Department of Urology, The Jikei University School of Medicine, Minato-ku, Tokyo Japan; 3 Department of Urology, Hamamatsu University School of Medicine, Hamamatsu, Shizuoka Japan; 4 Department of Urology, Fujita Health University School of Medicine, Toyoake, Aichi Japan; 5 Department of Urology, Osaka Medical and Pharmaceutical University, Takatsuki, Osaka Japan; 6 Department of Urology, Kawasaki Medical School, Kurashiki, Okayama Japan; 7 Department of Urology, Okayama University Graduate School of Medicine, Dentistry and Pharmaceutical Sciences, Okayama, Okayama Japan

**Keywords:** Metastatic renal cell carcinoma, Immune checkpoint inhibitors, Host-related factors, Prognosis, Combination therapy

## Abstract

**Background:**

Immune checkpoint inhibitor (ICI)-based combination therapies, including immune checkpoint inhibitor plus immune checkpoint inhibitor (IO + IO) and immune checkpoint inhibitor plus tyrosine kinase inhibitor (IO + TKI) regimens, have significantly improved outcomes in metastatic renal cell carcinoma (mRCC). However, the prognostic impact of host-related factors in patients receiving these therapies remains unclear.

**Methods:**

We retrospectively analyzed 493 patients with mRCC treated with IO+IO (*n* = 205) or IO+TKI (*n* = 288) as first-line therapy. Survival outcomes and treatment responses were evaluated according to host-related factors using Cox proportional hazards models.

**Results:**

In the IO+IO cohort, multiple host-related factors were independently associated with worse overall survival, including BMI < 25 kg/m^2^ (HR 2.280, *p* = 0.007), dyslipidemia (HR 2.079, *p* = 0.019), PPI use (HR 1.605, *p* = 0.039), and opioid use (HR 2.919, *p* = 0.008). In contrast, in the IO+TKI cohort, only IMDC poor risk (HR 4.169, *p* < 0.001) and opioid use (HR 3.328, *p* < 0.001) were significantly associated with overall survival. Host-related factors showed limited associations with objective response and disease control rates in both cohorts.

**Conclusion:**

The prognostic impact of host-related factors differs according to treatment regimen in mRCC. These findings suggest that host immune and metabolic conditions play a more prominent role in immune-driven IO+IO therapy, whereas tumor-related factors may predominate in IO+TKI therapy.

**Supplementary Information:**

The online version contains supplementary material available at 10.1007/s10147-026-03075-2.

## Introduction

Immune checkpoint inhibitor (ICI)-based combination therapies, including immune checkpoint inhibitor plus immune checkpoint inhibitor (IO + IO) and immune checkpoint inhibitor plus tyrosine kinase inhibitor (IO + TKI), have become the standard first-line treatment for metastatic renal cell carcinoma (mRCC). Several phase III clinical trials have demonstrated significant improvements in survival outcomes with these regimens compared with sunitinib monotherapy, establishing a new therapeutic era for mRCC management [1–3]. However, substantial heterogeneity in clinical outcomes remains even among patients within the same International Metastatic RCC Database Consortium (IMDC) risk category.

The IMDC risk model is widely used for prognostic stratification in mRCC, incorporating clinical and laboratory parameters such as performance status, anemia, hypercalcemia, neutrophilia, thrombocytosis, and time from diagnosis to systemic therapy [4]. Although this model reflects tumor burden and systemic inflammatory status, it may not fully capture host-related factors that influence treatment outcomes in the era of immunotherapy.

Recent studies have suggested that host-related factors, including body mass index (BMI) and metabolic comorbidities, may influence the efficacy of ICI therapy [5, 6]. In particular, an association between higher BMI and improved outcomes during ICI therapy, known as the “obesity paradox,” has been reported in several malignancies including RCC [7–9]. In addition, metabolic disorders such as dyslipidemia, diabetes mellitus, and cardiovascular disease may affect the tumor immune microenvironment through chronic systemic inflammation and metabolic dysregulation [10, 11].

Furthermore, concomitant medications such as proton pump inhibitors (PPI), antibiotics, and opioids have recently been reported to influence the outcomes of immunotherapy [12–14]. These medications may alter the gut microbiota and immune responses, and they may also reflect underlying comorbidities or symptom burden.

Importantly, the influence of host-related factors may not necessarily be reflected in early tumor response parameters such as objective response rate (ORR), but rather in long-term outcomes such as progression-free survival (PFS) and overall survival (OS). Moreover, because IO+IO and IO+TKI therapies differ in their mechanisms of action and tumor microenvironment modulation, the impact of host-related factors may vary depending on the treatment regimen.

Therefore, in the present study, we evaluated host-related factors—including BMI, metabolic comorbidities, and concomitant medications—in patients with mRCC receiving IO+IO or IO+TKI as first-line therapy. We investigated their association with clinical outcomes and assessed whether their prognostic impact differs according to treatment regimen.

## Materials and methods

We retrospectively analyzed the clinical database of 493 patients with mRCC who received IO+IO or IO+TKI as first-line therapy between January 2018 and December 2024 at multiple Japanese institutions, including The Jikei University School of Medicine, Okayama University Graduate School of Medicine, Dentistry and Pharmaceutical Sciences, Osaka Medical and Pharmaceutical University, Kindai University Faculty of Medicine, Fujita Health University School of Medicine, and their affiliated hospitals. Patients with incomplete medical records or missing information regarding treatment efficacy, such as imaging data or follow-up data, were excluded from the analysis. The study protocol was approved by the Institutional Review Board of the principal institution (Osaka Medical and Pharmaceutical University, Osaka, Japan; approval number: RIN750–2571). Because of the retrospective nature of the study, the requirement for informed consent was waived. The primary endpoint of this study was OS. Secondary endpoints included PFS and ORR. PFS was defined as the time from initiation of first-line therapy to disease progression or death from any cause. OS was defined as the time from initiation of first-line therapy to death from any cause.

Tumor responses were evaluated using the Response Evaluation Criteria in Solid Tumors (RECIST) version 1.1. ORR was defined as the proportion of patients achieving complete response (CR) or partial response (PR). Disease control rate (DCR) was defined as the proportion of patients achieving CR, PR, or stable disease (SD).

PPI use was defined as any documented use within the 30 days prior to and including the day of ICI initiation. Detailed information regarding the duration and indication of PPI use was not consistently available.

Comorbidities were defined based on documented medical history in the medical records. Dyslipidemia was defined as a documented medical history of dyslipidemia. BMI was analyzed as a host-related factor. For the primary analysis, patients were categorized into two groups according to BMI (< 25 vs. ≥ 25 kg/m^2^). Additional exploratory analyses were performed using three BMI categories: <18.5, 18.5–<25, and ≥ 25 kg/m^2^. A BMI of < 18.5 kg/m^2^ was used as a conventional definition of underweight, while a BMI of ≥ 25 kg/m^2^ was used to define obesity.

Continuous variables, including age, were compared using the Mann–Whitney U test. Categorical variables, including sex, IMDC risk classification, histology, metastatic sites, and comorbidities, were compared using the chi-square test or Fisher’s exact test, as appropriate. Survival outcomes were estimated using the Kaplan–Meier method and compared using the log-rank test. Cox proportional hazards regression analysis was performed to identify prognostic factors associated with survival outcomes. All variables included in the multivariable model were selected based on clinical relevance and prior literature. Variables with clinical relevance were included regardless of univariate significance. Statistical analyses were conducted using EZR (Saitama Medical Center, Jichi Medical University, Saitama, Japan), a graphical user interface for R (R Foundation for Statistical Computing, Vienna, Austria). The significance threshold was set at *p* < 0.05.

## Results

A total of 493 patients with mRCC were included in the analysis, of whom 205 and 288 received IO+IO and IO+TKI therapy as first-line treatment, respectively (Table 1). Most baseline characteristics were comparable between the two treatment groups, although IMDC risk classification and the proportion of patients with bone metastasis differed significantly.

**Table 1:** Patients’ characteristics

	IO+IO (n = 205)	IO+TKI (n = 288)	p-value
Median age (range)	69 (25–87)	71 (26–89)	0.133
Sex, n (%)			0.41
Male	163 (79.5)	214 (74.3)	
Female	42 (20.5)	74 (25.7)	
IMDC risk group, n (%)			< 0.001
Favorable	0 (0)	69 (24.0)	
Intermediate	122 (59.5)	152 (52.8)	
Poor	83 (40.5)	67 (23.3)	
Histology, n (%)			0.47
Clear cell	141 (68.8)	219 (76.0)	
Non-clear cell	38 (18.5)	40 (13.9)	
Unknown	26 (12.7)	29 (10.1)	
Metastatic site, n (%)			
Lung	124 (60.5)	162 (56.3)	1.000
Lymph node	77 (37.6)	79 (27.4)	1.000
Liver	23 (11.2)	33 (11.5)	0.384
Bone	47 (22.9)	75 (26.0)	0.039
Brain	10 (4.9)	6 (2.1)	1.000
Host-related factors, n (%)			
BMI category			0.271
< 25 kg/m^2^	161 (78.5)	198 (68.8)	
≥ 25 kg/m^2^	44 (21.5)	90 (31.3)	
Hemodialysis	17 (8.3)	14 (4.9)	0.604
Autoimmune disease	8 (3.9)	14 (4.9)	1.000
ASCVD	30 (14.6)	31 (10.8)	0.341
Dyslipidemia	21 (10.2)	39 (13.5)	1.000
PPI use	62 (30.2)	64 (22.2)	0.469
Opioid use	14 (6.8)	21 (7.3)	0.071

*IO* immune checkpoint inhibitor–based therapy,* TKI* tyrosine kinase inhibitor,* IMDC* international metastatic renal cell carcinoma database consortium,* BMI* body mass index,* ASCVD* atherosclerotic cardiovascular disease,* PPI* proton pump inhibitor

**Fig. 1:**
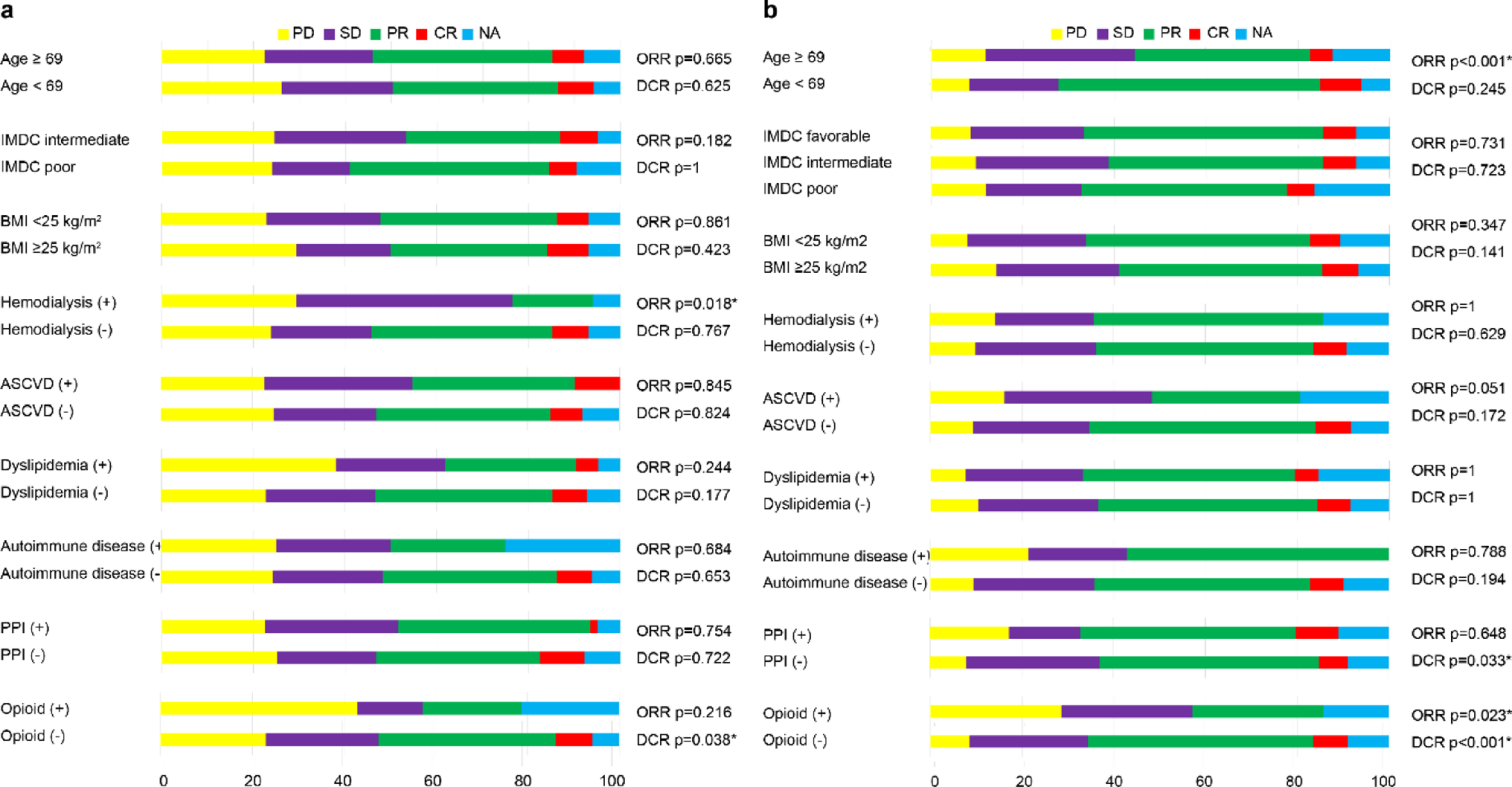
Treatment responses according to host-related factors in patients receiving first-line IO+IO (**a**) and IO+TKI (**b**). Stacked bar charts show best overall response (CR, PR, SD, PD, and NA) stratified by opioid use, BMI, and dyslipidemia. In the IO+IO cohort, opioid use was associated with a significantly lower disease control rate (DCR; *p* = 0.038), with no significant difference in overall response rate (ORR). In the IO+TKI cohort, opioid use was associated with significantly lower ORR (*p* = 0.023) and DCR (*p* < 0.001). No significant associations were observed for BMI or dyslipidemia

The association between host-related factors and treatment response (ORR and DCR) was evaluated in both cohorts (Fig. 1). In the IO+IO cohort, most host-related factors—including age, IMDC risk group, BMI, ASCVD, dyslipidemia, autoimmune disease, and PPI use—were not significantly associated with treatment response. However, patients receiving hemodialysis were associated with a significantly lower ORR compared with those without hemodialysis (*p* = 0.018). In addition, opioid use was associated with a significantly lower DCR (*p* = 0.038), whereas no significant difference in ORR was observed (*p* = 0.216). In the IO+TKI cohort, most host-related factors were also not significantly associated with treatment response. However, opioid use was associated with both lower ORR (*p* = 0.023) and lower DCR (*p* < 0.001). In addition, PPI use was associated with a lower DCR (*p* = 0.033). Overall, host-related factors showed limited associations with treatment response in both cohorts.

**Fig. 2:**
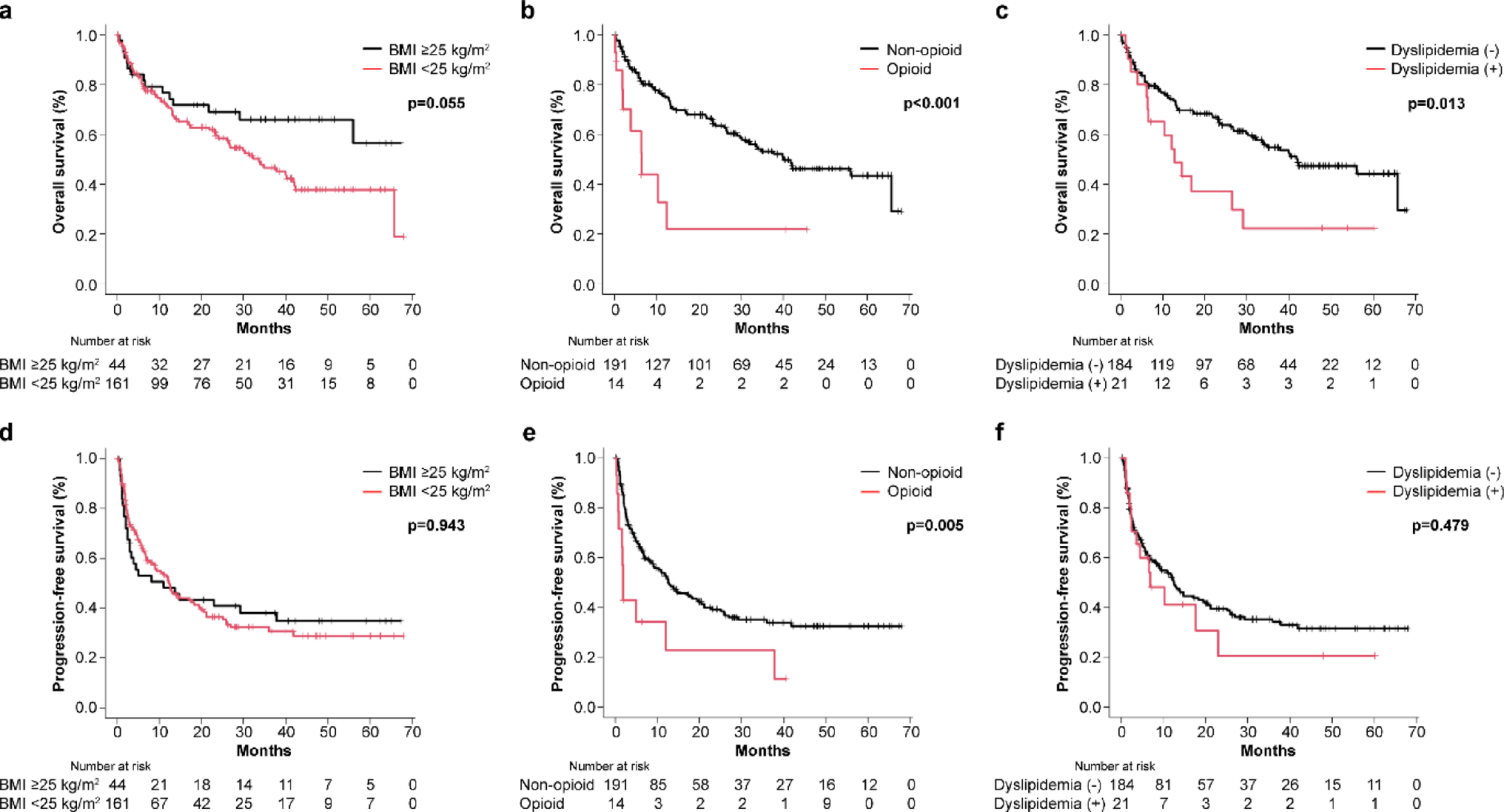

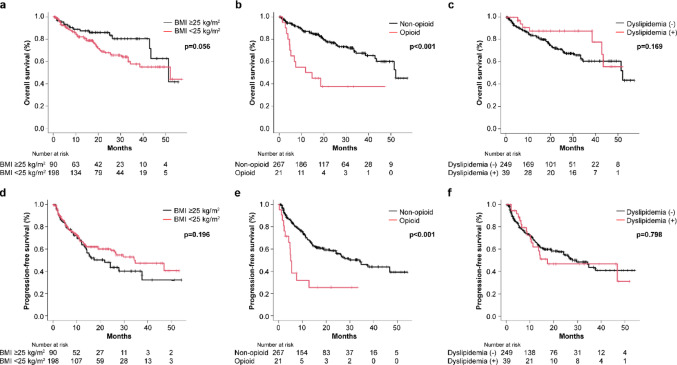
Kaplan–Meier curves for progression-free survival (PFS) and overall survival (OS) according to host-related factors in patients receiving first-line IO+IO (**a**) and IO+TKI (**b**) therapy. In the IO+IO cohort (**a**), opioid use was significantly associated with shorter PFS (*p* = 0.005) and OS (*p* < 0.001). Dyslipidemia was associated with worse OS (*p* = 0.013), whereas BMI showed no significant association with PFS and a borderline association with OS (*p* = 0.055). In the IO+TKI cohort (**b**), opioid use was significantly associated with shorter PFS and OS (both *p* < 0.001), whereas BMI and dyslipidemia were not associated with survival outcomes

**Fig. 3:**
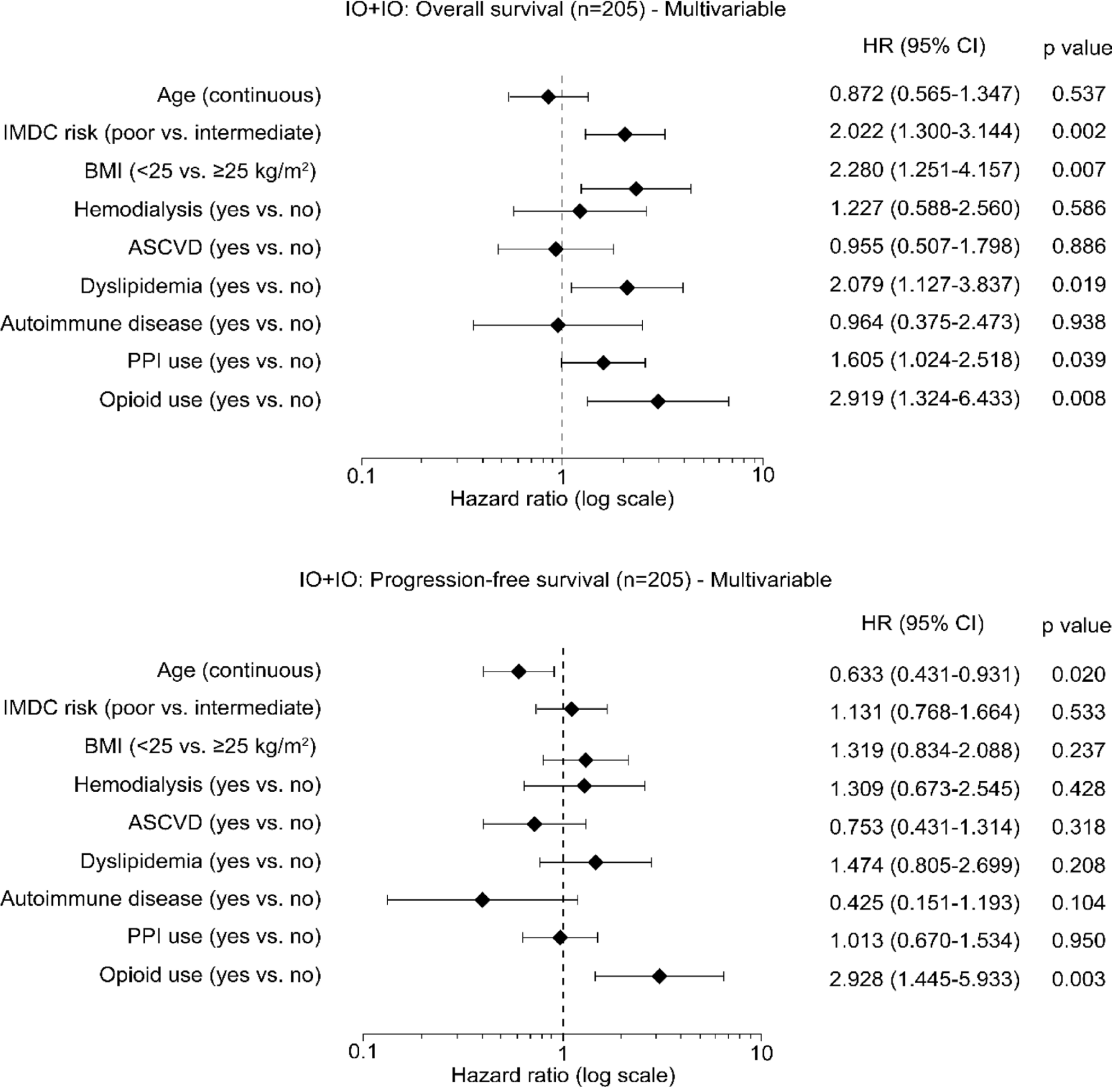

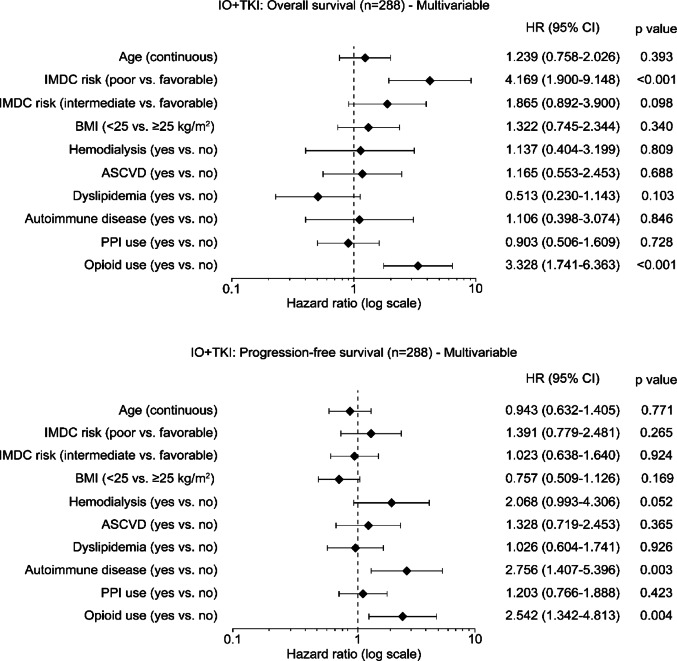
Multivariable Cox regression analysis for progression-free survival (PFS) and overall survival (OS) in patients receiving first-line IO+IO (**a**) and IO+TKI (**b**). In the IO+IO cohort (**a**), age (*p* = 0.020) and opioid use (*p* = 0.003) were independently associated with shorter PFS. For OS, IMDC poor risk (*p* = 0.002), BMI < 25 kg/m^2^ (*p* = 0.007), dyslipidemia (*p* = 0.019), PPI use (*p* = 0.039), and opioid use (*p* = 0.008) were independently associated with worse outcomes. In the IO+TKI cohort (**b**), opioid use (*p* = 0.004) and autoimmune disease (*p* = 0.003) were independently associated with shorter PFS. For OS, IMDC poor risk (vs. favorable, *p* < 0.001) and opioid use (*p* < 0.001) were independently associated with worse outcomes

Kaplan–Meier (KM) analysis in the IO+IO cohort showed that opioid use was significantly associated with shorter PFS (log-rank *p* = 0.005), whereas no significant differences were observed for BMI or dyslipidemia (Fig. 2a). Multivariable Cox regression analysis identified age and opioid use as independent predictors of shorter PFS (age: HR 0.633, 95% CI 0.431–0.931, *p* = 0.020; opioid use: HR 2.928, 95% CI 1.445–5.993, *p* = 0.003) (Fig. 3a). For OS in the IO+IO cohort, KM analysis demonstrated that opioid use and dyslipidemia were significantly associated with worse OS (log-rank *p* < 0.001 and *p* = 0.013, respectively), while BMI < 25 kg/m^2^ showed a borderline association (*p* = 0.055) (Fig. 2a). Additional analyses based on BMI categories (< 18.5, 18.5–<25, and ≥ 25 kg/m^2^) for OS and PFS in both the IO+IO and IO+TKI cohorts are presented in the Supplementary Materials (Supplementary Figs. S1 and S2). No statistically significant differences among the BMI groups were observed in either cohort (IO + IO: OS *p* = 0.149, PFS *p* = 0.805; IO + TKI: OS *p* = 0.139, PFS *p* = 0.316). Multivariable analysis confirmed IMDC poor risk (HR 2.022, 95% CI 1.300–3.144, *p* = 0.002), BMI < 25 kg/m^2^ (HR 2.280, 95% CI 1.251–4.157, *p* = 0.007), dyslipidemia (HR 2.079, 95% CI 1.127–3.837, *p* = 0.019), PPI use (HR 1.605, 95% CI 1.024–2.518, *p* = 0.039), and opioid use (HR 2.919, 95% CI 1.324–6.433, *p* = 0.008) as independent predictors of worse OS (Fig. 3a).

In the IO+TKI cohort, KM analysis showed that opioid use was significantly associated with shorter PFS (log-rank *p* < 0.001), whereas BMI and dyslipidemia were not associated with PFS (Fig. 2b). Multivariable Cox regression analysis identified opioid use (HR 2.542, 95% CI 1.342–4.813, *p* = 0.004) and autoimmune disease (HR 2.756, 95% CI 1.407–5.396, *p* = 0.003) as independent predictors of shorter PFS, with a borderline association observed for hemodialysis (HR 2.068, 95% CI 0.993–4.306, *p* = 0.052) (Fig. 3b). For OS in the IO+TKI cohort, KM analysis demonstrated that opioid use was significantly associated with worse OS (log-rank *p* < 0.001), while BMI < 25 kg/m^2^ showed a borderline association (*p* = 0.056), and no significant difference was observed for dyslipidemia (Fig. 2b). Multivariable analysis identified IMDC poor risk (HR 4.169, 95% CI 1.900–9.148, *p* < 0.001) and opioid use (HR 3.328, 95% CI 1.741–6.363, *p* < 0.001) as independent predictors of worse OS (Fig. 3b). Overall, host-related factors showed a broader and stronger association with overall survival in the IO+IO cohort, whereas their impact was more limited in the IO+TKI cohort.

## Discussion

In this multicenter study, we demonstrated that the prognostic impact of host-related factors differs substantially between IO+IO and IO+TKI regimens in patients with metastatic renal cell carcinoma (mRCC). Host-related factors, including BMI, dyslipidemia, PPI use, and opioid use, were significantly associated with overall survival in the IO+IO cohort, whereas similar associations were not observed in the IO+TKI cohort. These findings indicate a regimen-dependent impact of host-related factors, with a broader influence observed in IO+IO therapy compared with IO+TKI therapy. Furthermore, host-related factors may play a more prominent role in immune-driven therapies than in VEGFR inhibitor combination therapies.

Obesity is known to be associated with chronic systemic inflammation and immune dysregulation. Recently, the association between BMI and outcomes of immune checkpoint inhibitor therapy, known as the “obesity paradox,” has been reported, suggesting that obese patients may experience improved outcomes with ICI treatment. Previous studies have demonstrated improved clinical outcomes of ICIs in obese patients [7–9].

In the present study, BMI was significantly associated with overall survival in the IO+IO cohort, consistent with these previous reports, whereas no such association was observed in the IO+TKI cohort. These findings suggest that the “obesity paradox” may be more relevant in immune-driven therapies than in combination therapy with VEGFR inhibitors.

IO+IO therapy primarily relies on activation of antitumor immune responses and may therefore be more susceptible to host immune conditions associated with obesity-related inflammation. Indeed, obesity has been reported to influence PD-1 expression and T-cell function, which may affect the efficacy of immune checkpoint inhibitor therapy [15]. In contrast, IO+TKI therapy includes anti-angiogenic agents that can induce tumor vascular normalization and enhance immune cell infiltration within the tumor microenvironment [16]. These effects may shift the balance toward tumor-related factors and attenuate the relative impact of host-related factors, although this remains speculative.

Consistent with this concept, clinical observations have shown that treatment efficacy varies according to metastatic site, likely reflecting differences in the tumor microenvironment and biological behavior rather than host-related factors alone. Previous studies have demonstrated that IO+TKI may provide greater benefit in certain metastatic sites, such as liver metastases, where an immunosuppressive microenvironment may limit the effectiveness of immune-based therapies [17]. In the present study, hemodialysis was associated with a lower ORR only in the IO+IO cohort; however, no significant association was observed with DCR, PFS, or OS. These findings suggest that although tumor shrinkage may be less frequently observed in patients undergoing hemodialysis, disease stabilization and long-term outcomes may not be compromised. Because the number of patients receiving hemodialysis in this cohort was limited, these results should be interpreted with caution.

Dyslipidemia represents another metabolic abnormality that may influence immune function and the tumor microenvironment. In the present study, dyslipidemia was associated with overall survival in the IO+IO cohort but not in the IO+TKI cohort. Previous studies have suggested that lipid metabolism can modulate immune responses and tumor immunity [10, 11]. Host metabolic abnormalities, including dyslipidemia, may reflect chronic systemic inflammation and altered immune competence [10]. Metabolic conditions affecting T-cell–mediated immunity may have a greater impact in treatment settings primarily driven by immune activation. Dyslipidemia may reflect a host metabolic and immune milieu unfavorable for durable benefit from IO + IO, potentially contributing to inferior long-term survival outcomes in this population. Our findings are consistent with these reports and further support the concept that host metabolic status may influence immune responses during immunotherapy. Because IO+IO therapy primarily relies on immune activation, host metabolic conditions such as dyslipidemia may have a greater impact on treatment outcomes in this setting.

Interestingly, BMI and dyslipidemia were not significantly associated with ORR or DCR in this study. Previous reports have suggested that early tumor response does not necessarily correlate with long-term survival outcomes in patients receiving immune checkpoint inhibitors [1, 18]. This temporal discrepancy suggests that host-related factors may exert a greater influence on long-term survival than on early tumor response.

Proton pump inhibitors are widely used medications that suppress gastric acid secretion, and their potential influence on immune checkpoint inhibitor therapy has recently attracted increasing attention [6, 19]. In the present study, PPI use was associated with worse overall survival in the IO+IO cohort but not in the IO+TKI cohort [20]. PPIs have been reported to alter the gut microbiome, and such alterations may influence the efficacy of immune checkpoint inhibitors [12, 19]. Our findings are consistent with previous reports suggesting that PPI use may negatively affect outcomes in patients receiving immunotherapy. Because IO+IO therapy relies primarily on immune activation, it may be more susceptible to the effects of host-related factors such as alterations in the gut microbiome. In our cohort, PPI exposure was defined within the 30 days prior to and including the day of ICI initiation, and we were unable to assess the impact of the duration of PPI use due to the retrospective nature of the dataset and the lack of detailed longitudinal medication records. Unlike antibiotics, for which the timing of exposure has been relatively well characterized [19], there is currently no established consensus regarding the optimal time window for defining PPI exposure, and its effects on the gut microbiota are likely cumulative and dependent on the duration of use [12]. Therefore, further studies evaluating the impact of short-term versus long-term PPI use are warranted.

Patients with autoimmune disease were significantly associated with worse progression-free survival in the IO+TKI cohort but not in the IO+IO cohort. Previous studies have reported that patients with autoimmune diseases may experience altered immune responses and immune-related adverse events during immune checkpoint inhibitor therapy [21, 22]. This discrepancy suggests that the clinical impact of autoimmune disease may differ depending on treatment context.

These results should be interpreted with caution given the limited number of patients with autoimmune disease. However, no association with overall survival was observed, and the relatively small sample size and the potential influence of subsequent therapies may have affected this result.

Among all host-related factors examined, opioid use consistently demonstrated the strongest and most reproducible association with adverse outcomes across both treatment regimens. Opioid use was associated with significantly worse overall survival and progression-free survival in both the IO+IO and IO+TKI cohorts, and was also associated with a lower disease control rate. Since patients receiving opioids are likely to have a higher tumor burden and more advanced disease, it is reasonable that they have a worse prognosis. Clinical studies have also reported an association between opioid use and poorer outcomes in patients receiving immune checkpoint inhibitor therapy. In addition, opioids have been reported to exert immunosuppressive effects, including inhibition of T-cell function and cytokine production, which may attenuate antitumor immune responses [23]. These findings suggest that opioid use may represent not only a surrogate of disease burden but also a potential modifier of antitumor immunity.

Several limitations of this study should be acknowledged. First, this study was retrospective and the possibility of selection bias cannot be completely excluded. Second, although this study included data from multiple institutions, the sample size was relatively limited in each cohort. Third, detailed information regarding the indication and duration of concomitant medications was not fully available. Fourth, unmeasured confounders such as nutritional status may have influenced the observed associations.

From a clinical perspective, our findings suggest that host-related factors should be considered when selecting treatment regimens for patients with mRCC. Patients with unfavorable host-related conditions, such as low BMI or metabolic abnormalities, may derive greater benefit from IO+TKI therapy, which is less dependent on host immune status. In contrast, patients with more favorable host conditions may be better candidates for IO+IO therapy, which relies primarily on immune activation. These findings highlight the potential importance of incorporating host-related characteristics into personalized treatment decision-making.

In conclusion, the prognostic impact of host-related factors differs according to treatment regimen in patients with mRCC receiving immune-based combination therapy. Host-related factors were more strongly associated with long-term survival in the IO+IO cohort, whereas their impact was more limited in the IO+TKI cohort. These findings highlight the importance of incorporating host-related characteristics into treatment decision-making and prognostic stratification in the era of immune combination therapy.

## Supplementary Information

Below is the link to the electronic supplementary material.

Supplementary Material 1 Kaplan–Meier curves for overall survival (OS) according to BMI categories (< 18.5, 18.5–<25, and ≥ 25 kg/m^2^ in the IO+IO (a) and IO+TKI (b) cohorts. The log-rank test was used to compare survival between groups.

Supplementary Material 2 Kaplan–Meier curves for progression-free survival (PFS) according to BMI categories (< 18.5, 18.5–<25, and ≥ 25 kg/m^2^) in the IO+IO (a) and IO+TKI (b) cohorts. The log-rank test was used to compare survival between groups.

## Data Availability

The datasets generated and/or analyzed during the current study are available from the corresponding author upon reasonable request.
