# Supplementary figures and images for: Differential impact of host-related factors on survival outcomes between IO+IO and IO+TKI in metastatic renal cell carcinoma: a multicenter retrospective study
Source: Int J Clin Oncol. 2026 Jun 11;31(8):1658–67. doi: 10.1007/s10147-026-03075-2 (PMC13401547; doi:10.1007/s10147-026-03075-2)

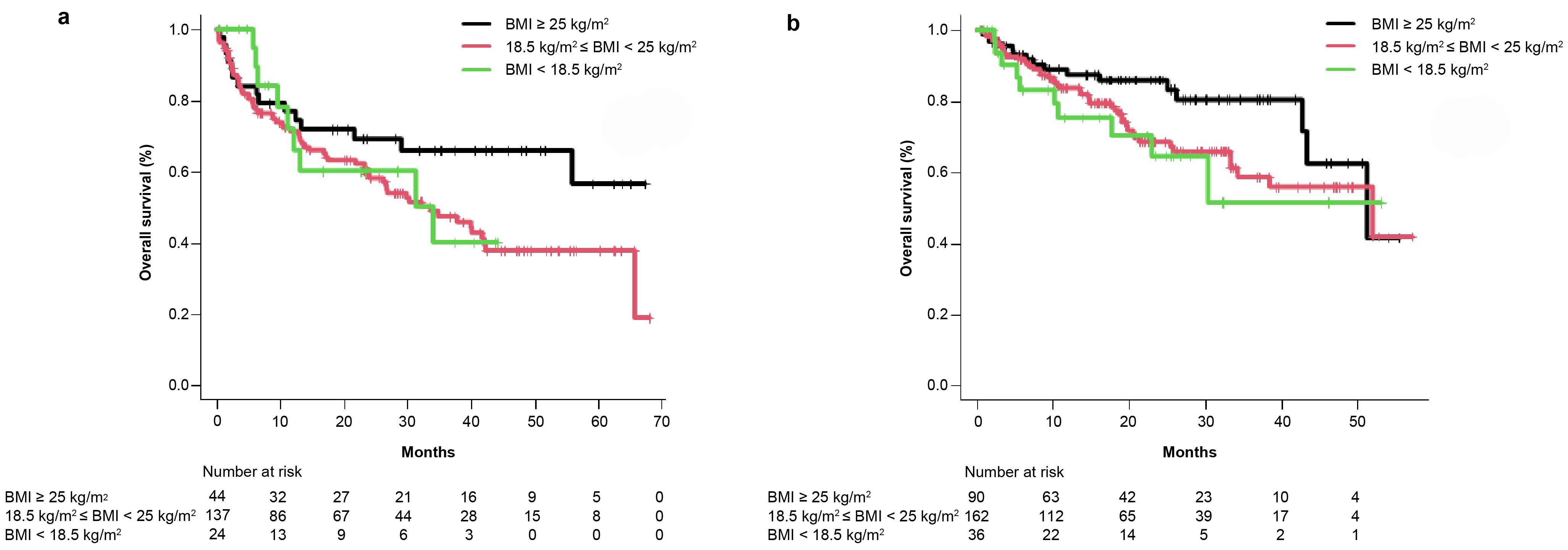

Supplement: Supplementary file 1 — Supplementary Material 1 Kaplan–Meier curves for overall survival (OS) according to BMI categories (< 18.5, 18.5–<25, and ≥ 25 kg/m2 in the IO+IO (a) and IO+TKI (b) cohorts. The log-rank test was used to compare survival between groups. [file 10147_2026_3075_MOESM1_ESM.tif]

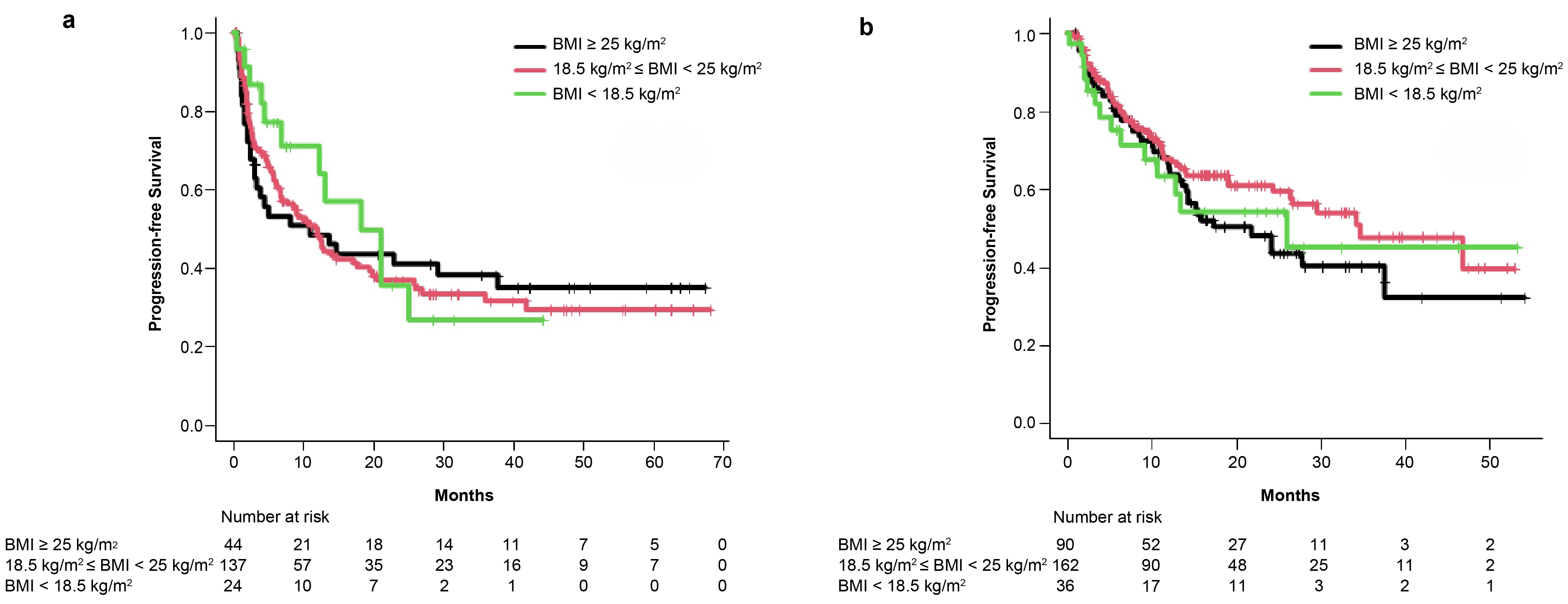

Supplement: Supplementary file 2 — Supplementary Material 2 Kaplan–Meier curves for progression-free survival (PFS) according to BMI categories (< 18.5, 18.5–<25, and ≥ 25 kg/m2) in the IO+IO (a) and IO+TKI (b) cohorts. The log-rank test was used to compare survival between groups. [file 10147_2026_3075_MOESM2_ESM.tif]
